# Economic abuse and its associations with symptoms of common mental disorders among women in a cross-sectional survey in informal settlements in Mumbai, India

**DOI:** 10.1186/s12889-021-10904-8

**Published:** 2021-05-01

**Authors:** Suman Kanougiya, Nayreen Daruwalla, Lu Gram, Apoorwa Deepak Gupta, Muthusamy Sivakami, David Osrin

**Affiliations:** 1 Program on Prevention of Violence Against Women and Children, SNEHA, Mumbai, Maharashtra 400017 India; 2 School of Health Systems Studies (SHSS), Tata Institute of Social Sciences (TISS), Mumbai, Maharashtra, India; 3 Institute for Global Health, University College London, London, WC1N IEH UK

**Keywords:** Economic abuse, Domestic violence, Common mental disorders, Depression, Anxiety, Suicidal ideation, India

## Abstract

**Background:**

Domestic violence takes a range of interconnected forms, of which economic abuse is common, but less studied than others. We examine the prevalence of economic abuse, its determinants, and its association with symptoms of depression, anxiety, and suicidal ideation.

**Methods:**

Our cross-sectional survey in informal settlement areas in Mumbai, India, asked women aged 18–49 years 15 questions about acquisition, use, and maintenance of economic resources, demographic and socioeconomic factors, and physical, sexual, and emotional violence. We administered the Patient Health Questionnaire 9 (PHQ-9) and Generalised Anxiety Disorder 7 (GAD-7) scales and asked about suicidal thinking. Determinants of economic abuse and its associations with positive screens for depression and anxiety were explored in univariable and multivariable logistic regression models.

**Results:**

Of 4906 ever-married women respondents, 23% reported at least one form of economic abuse by either an intimate partner or another family member. The commonest were denial of property rights (10%), not being trusted with money (8%), and coercive appropriation of belongings (7%). Economic abuse was more commonly reported by widowed, separated, or divorced women than by married women (aOR 12.4; 95% CI 6.4, 24.1), and when their partners used alcohol or drugs (aOR 1.4; 95% CI 1.2–1.7). Women had greater odds of reporting economic abuse if they had suffered emotional (aOR 6.3; 95% CI 5.0–7.9), physical (aOR 1.9; 95% CI 1.4–2.6), or sexual violence (aOR 5.4; 95% CI 3.6–8.1) in the preceding 12 months. Economic abuse was independently associated with positive screens for moderate-severe depression (aOR 2.6; 95% CI 2.0–3.4), anxiety (aOR 2.7; 95% CI 1.9–3.8), and suicidal ideation (aOR 2.2; 95% CI 1.5–3.1). The odds of anxiety and depression increased with each additional form of economic abuse.

**Discussion:**

To our knowledge, this is the first community-based study in India of the prevalence of economic abuse and its associations with symptoms of common mental disorders. It provides empirical support for the idea that economic abuse is at least as harmful to women’s mental health as physical violence. Surveys should include questions on economic abuse and prevention and intervention strategies need to help survivors to understand its forms.

## Background

Violence against women occurs across all regions, societies, and cultures [1]. It is a serious public health concern which affects the physical [2], reproductive [3], mental [4, 5], and social wellbeing of more than one-third of women [6]. It is also widely underreported [7]. Domestic violence takes a range of forms. The most often measured are physical and sexual violence [8, 9], but the importance of emotional or psychological violence as an insidious cause of harm to women’s wellbeing is becoming apparent [10, 11]. Global considerations have focused on intimate partner violence, a form of domestic violence described as “any behaviour by a current or former male intimate partner within the context of marriage, cohabitation or any other formal or informal union, that causes physical, sexual or psychological harm” [6]. In South Asia, considerations of violence by perpetrators within the family extend beyond intimate partners [12], not only to sexual violence by perpetrators other than partners [6, 13], but to forms of violence perpetrated by other family members. India’s Protection of Women from Domestic Violence Act, 2005, defines domestic violence as occurring within a domestic relationship: “a relationship between two persons who live or have, at any point of time, lived together in a shared household, when they are related by consanguinity, marriage, or through a relationship in the nature of marriage, adoption or are family members living together as a joint family.” [14].

Domestic violence is therefore a composite of intimate partner violence and violence by others in the context of the home. We follow this broader definition in considering economic abuse [9], a means of power and control within domestic relationships [15] whose dimensions have recently gained attention [16, 17]. Economic abuse overlaps with emotional violence in its use of coercive control [18–20], but is increasingly recognised as a category of violence in itself [21, 22]. Its reduction is essential to achieving at least five of the 17 United Nations Sustainable Development Goals.

A developing consensus defines economic abuse as control over a person’s ability to obtain, use, or sustain access to economic resources in a manner which diminishes the victim’s capacity to support herself, threatens her economic security and potential for self-sufficiency, or forces her to depend on perpetrators financially [21, 23, 24]. The definition admits a broad range of economic acts; for example, preventing access to property, disrupting employment (preventing employment outside the home or preventing an employed person from achieving their working hours [25, 26]), depleting savings and assets or generating expenditure and debt [25], controlling or destroying money and resources, and eviction from the home. Limiting the survivor’s access to resources creates a vicious circle in which their capacity to change the abusive situation is compromised [27, 28].

Estimates of prevalence vary with location, source, and methods [29]. Most estimates of lifetime prevalence of economic abuse come from high-income countries: 3–5% in Canada from police reports and a social survey [30, 31], 15% from a survey in Australia [32], 12–15% from cohort studies in the United States [18, 33, 34], and 21% from a nationally representative survey in the United Kingdom [35]. Estimates in middle-income settings include 7% from a Demographic and Health Survey in the Philippines [36], 28% from a village census in Vietnam [37], 45% from national surveys among Palestinian women [38], and 62% in a survey in rural Bangladesh [29].

India is a signatory to the Convention on the Elimination of all Forms of Discrimination Against Women and the International Covenant on Economic, Social, and Cultural Rights. India’s Protection of Women from Domestic Violence Act, 2005 aims to protect women’s rights guaranteed under the constitution and applies to violence of any kind occurring within the family. It characterises economic abuse as a form of domestic violence, an advance given that it is often not considered or recognised beyond dowry violence [39]. Studies on economic abuse in India have reported lifetime prevalence ranging from 10% in a community-based study in Haryana [40], 11% among both men and women attending a general hospital in Gujarat [41], 37% among women outpatients at an Urban Health Centre in Punjab [42], and 89% in a four-site study [43].

Economic abuse reduces employment opportunities and stability, diminishes resources for survival such as housing and money, lowers the standard of living, adversely affects childcare and social capital and diminishes economic self-sufficiency and self-efficacy. Marital dependency and Interdependence theories suggest that women are financially, educationally, and occupationally dependent on their male partners. Economic dependence is a significant obstacle for a woman who wishes to leave an abusive partner and limits her ability to end a violent relationship. Women who escape such relationships are often impoverished, and economic abuse may continue even after they have left. Economic and interpersonal relationship difficulties are associated with the development of depression and anxiety, and suicidal ideation [5, 44–50]. Women who experience economic abuse may suffer similar mental health consequences to those caused by violent abuse, although the link between economic abuse and common mental disorders has not been well identified [17, 25, 51, 52].

Three aspects of economic abuse of women have gained attention in recent years: the need to measure it [16], the need to understand its prevalence [16, 53], and the need to understand its effects on women’s mental health [17, 50]. There have been few studies of the association between economic abuse and common mental disorder, and none in India [34]. We aimed, therefore, to examine the prevalence of economic abuse, its determinants, and its association with symptoms of depression, anxiety, and suicidal ideation in informal settlements in Mumbai.

## Methods

### Setting

Urban informal settlements are characterised by overcrowding and unsanitary and unhealthy living conditions [54]. Residents face insecure tenure, limited safe drinking water, sanitation, drainage, and solid waste management, poor internal and approach roads, limited street lighting, and poor quality of shelter [55]. Data for our study come from a survey done before implementing a community-based intervention to address violence against women in informal settlements in Mumbai [56]. The non-government organisation SNEHA (Society for Nutrition, Education and Health Action) has run a program focusing on primary, secondary, and tertiary prevention of violence for 20 years. The primary beneficiaries of the program are residents of informal settlements. Primary prevention is addressed through a combination of community group activities and resulting individual voluntarism. Secondary prevention includes local crisis response and psychological first aid by community organisers and referral to centres which provide counselling, legal, and psychotherapeutic support, with links to the police and medical, shelter, and social service providers. Tertiary prevention is provided primarily through referral to psychiatric and legal services.

### Design

Data come from a cross-sectional systematic random sample survey in 50 equally-sized clusters of roughly 500 residential households in two large informal settlement areas. From a random starting point in each cluster, alternate households were enumerated without replacement until we had collected information from 100 women aged 18–49 years. When more than one potential respondent was available in a household, an algorithm led the investigators to select the youngest disabled, youngest married, or youngest unmarried woman. We did this to ensure representation of younger married women with disability who may be more vulnerable to domestic violence [57].

### Data collection

Between 5th December 2017 and 28th March 2019, 5277 households were approached for the survey. A fuller description of data collection is available elsewhere [58]. Briefly, 16 women interviewers with graduate education and 3 months of training mapped the study areas and enumerated household residents. After explaining the study, interviewers allowed women time to consider participation. They were supported by three field supervisors with a direct linkage to counselling services, available by phone at any time. In order to maintain privacy, interviews were arranged by advance appointment and avoided times when partners or children were likely to return from work or school. Women were interviewed at home or in a local community office if they preferred it. The interview began with general questions about demography, household residents, education, socioeconomic position, maternity, and health. If a family member, neighbour, or friend entered, the interviewer went back to asking questions about general health. If the person showed signs of staying, the interview was terminated and completed over up to three repeat visits. Gatekeepers and community members were briefed that SNEHA had planned to start work on violence against women in these areas and not only collect data. This would eventually help the families residing in these communities. As a result of the gatekeeper consent process, community members were aware that interviewers would be visiting people in their area and this limited intrusion. Interviewers used electronic tablets to enter information in a database in CommCare (www.dimagi.com).

### Variables

We used existing Hindi versions of scales where possible. If not, they were translated from English, piloted in two clusters external to the trial, amended, and back-translated. We screened for depression with the Patient Health Questionnaire 9 (PHQ-9) questionnaire [59] and anxiety with the Generalised Anxiety Disorder 7 (GAD-7) questionnaire [60, 61]. For both screens, questions referred to the last 2 weeks and each item was coded 0 (*not at all*), 1 (*several days*), 2 (*more than half the days*), or 3 (*nearly every day*). Both of these questionnaires are established and recommended in mental health research in India. A version of the PHQ-9 has been validated in 11 Indian languages [62], but local validation studies of the GAD-7 have been limited [63–66]. Our analyses used binary variables describing moderate or severe symptoms of depression and anxiety. A PHQ-9 score of 10–27 was taken as suggesting at least moderate depression [67], and a GAD-7 score of 10–21 as suggesting at least moderate anxiety. We asked two questions about suicidality: “In the past 12 months, did you ever consider attempting suicide?” and “in the past 12 months, did you ever attempt suicide?” [68]. Our analyses used a binary composite of both questions.

We asked respondents about their experience of violence perpetrated by intimate partners or other family members. We described the experience of economic abuse with binary responses to 15 individual questions based on programme experience in supporting survivors of violence, augmented by four focus group discussions with counsellors, community actors, and lawyers. Questions covered, over a woman’s lifetime, (1) denying her the right to property, (2) coercive appropriation of her belongings or (3) money or use of her bank account, (4) convincing her to make a loan that was not repaid, (5) selling her valuables without consent, (6) lying to her about employment or finances, (7) harassing her for not bringing natal money or property into the marital family or (8) coercing her to do so, (9) preventing her from seeking employment, (10) hiding money from her, (11) taking a loan without her consent, (12) gambling without her consent, (13) not trusting her with money, (14) keeping from having enough money, and (15) coercing her to hand over her income. The answers to each these questions could apply to either an intimate partner or another domestic perpetrator. They map onto an Economic Coercion Scale recently developed from work in Bangladesh, which includes 36 questions representing two domains: interference in acquisition of economic resources and interference in the use or maintenance of economic resources [29]. We summed these responses to generate a composite score describing the intensity of economic abuse, with values from 0 to 15.

Emotional violence was described by five questions, physical violence by nine, and sexual violence by four [58]. Women’s affirmative response to any of these questions—lifetime or past year—was described by a binary composite of physical violence, sexual violence, and emotional violence. Marital status was described by a categorical binary variable distinguishing married respondents from respondents who had been widowed, separated, or divorced. Socioeconomic position was described by quintiles of a standardised score derived from the first component of a principal components analysis of the ownership of 22 assets [69, 70].

Cronbach’s alpha indicated internal consistency for the PHQ-9 (α 0.86), GAD-7 (α 0.84), 15-items on economic abuse (α 0.86), nine items on physical abuse (α 0.83), four items on sexual abuse (α 0.76), and five items on emotional abuse (α 0.82).

### Sample size

Completion of 100 questionnaires in each of 50 clusters would yield a total sample of 5000. An estimate of prevalence from a cross-sectional sample of 4900 ever-married women in a population of 125,000 would have a precision of ~ 1–1.5%. Within this, and assuming a conservative intracluster correlation coefficient of 0.05, a comparison of two categories of a determinant for 100 respondents in each of 50 clusters would provide 80% power to detect a difference of 6% in prevalence estimates of 10–20%.

### Statistical analysis

We tabulated frequencies and proportions of demographic and socioeconomic variables, responses to questions about economic abuse, lifetime and 12-month experience of physical, sexual, and emotional violence, and depression, anxiety, and suicidal thoughts and action. We used cross-tabulation to examine associations between economic abuse, demographic and socioeconomic characteristics, and other forms of violence. We assessed the determinants of economic abuse in univariable and multivariable logistic regression models. We then examined the association of symptoms of common mental disorders with economic abuse in a series of univariable and multivariable logistic regression models with moderate or severe depression, moderate or severe anxiety, and suicidal thoughts or action in the last 12 months as dependent variables. Unadjusted models included economic abuse, emotional, physical, and sexual violence as exposures, followed by two adjusted models: the first including covariates for respondent age, education, religion, caste, socioeconomic asset quintile, respondent and husband employment, and respondent and husband drug or alcohol use, and the second adjusted for the same variables and also for other forms of violence. Finally, we examined the effect of increasing numbers of forms of economic abuse on moderate or severe depression, moderate or severe anxiety, and suicidal thoughts or action in the last 12 months. We adjusted the logistic regression models in the same way as above and then predicted marginal effects to illustrate the increase in the proportion of women with symptoms of common mental disorders for each unit increase in acts of economic abuse from 0 to 15. We modelled the log-odds of a positive screen for moderate or severe common mental disorder as a step function from 0 to 1 act of economic abuse, followed by a linear increase from 1 to 15 acts. We tested for non-linearity by fitting a quadratic term for the increase from 1 to 15. The analysis was carried out in STATA 15.0 (StataCorp LLC), and all estimates were adjusted for survey design.

## Results

Table 1 summarises characteristics of 4906 ever-married women respondents. Around 19% had had no schooling, and 38% had reached middle school. A quarter of women were in remunerated work—although 20% of these women earned less than INR 12,000 a year (USD 163)—and 98% of their partners were in remunerated work with a mean annual income of INR 172,383 (USD 2335). More than half identified as Hindu and of general caste. 12% said that they used alcohol or drugs, compared with 44% of their husbands.

**Table 1:** Characteristics of 4906 ever-married women in informal settlements in Mumbai

	Frequency	(%)
Marital status		
Currently married	4694	(96)
Widowed, separated, or divorced	212	(4)
Respondent age (years)		
18–25	1025	(21)
26–30	1421	(29)
31–36	1172	(24)
37–49	1288	(26)
Respondent education (school years)		
No education	938	(19)
Primary 1–5	846	(17)
Middle 6–8	1099	(22)
High 9–10	1105	(23)
Senior 11–12	533	(11)
Above 12	385	(8)
Children		
0	8	(< 1)
1	950	(22)
2	1666	(38)
3	1040	(24)
4+	704	(16)
Respondent in remunerated work	1182	(24)
Respondent monthly income (Indian Rupees)		
< 1000	233	(5)
1000–2999	303	(6)
3000–5999	279	(6)
6000+	322	(7)
Religion		
Hindu	2882	(59)
Muslim	1826	(37)
Other	198	(4)
Housing type		
Kachha (insubstantial)	336	(7)
Pukka (robust)	2518	(51)
Mixed	2052	(42)
Toilet type		
Private	836	(17)
Public	4368	(82)
Open defecation	2	(< 1)
Socioeconomic quintile		
1 poorest	969	(20)
2	936	(20)
3	934	(20)
4	933	(20)
5 least poor	935	(20)
Respondent uses alcohol or drugs	612	(12)
Husband age (years)		
18–19	14	(< 1)
20–29	917	(19)
30–39	2102	(44)
40–49	1370	(29)
50+	391	(8)
Husband in remunerated work	4686	(98)
Husband monthly income (Indian Rupees)		
< 10,000	1095	(24)
10,000-11,999	997	(21)
12,000-14,999	652	(14)
15,000+	1942	(41)
Husband uses alcohol or drugs	2100	(44)
All	4906	(100)

Table 2 summarises the prevalence of economic abuse, domestic violence, and screening for common mental disorders. Overall, 23% of women reported experiencing at least one of the 15 forms of economic abuse, with no missing observations. The commonest were that their property rights had been denied, or that belongings had been taken from them. Forms of violence other than economic abuse were also common, the commonest being emotional violence. Overall, 9% of women screened positive for moderate or severe depressive symptoms on the PHQ-9, 6% for anxiety on the GAD-7, and 6% had contemplated or attempted suicide in the last year.

**Table 2:** Prevalence of economic abuse, domestic violence, and symptoms of common mental disorder among 4906 ever-married women in informal settlements in Mumbai

	n	(%)
Lifetime economic abuse		
Denied the right to property	488	(10)
Not trusted with money	394	(8)
Belongings taken by force	330	(7)
Money hidden from respondent	277	(6)
Told lies about job or finances	263	(5)
Convinced to loan money and not repaid	255	(5)
Valuables sold without consent	243	(5)
Kept from having enough money	230	(5)
Harassed for not bringing natal family money or property	222	(4)
Prevented from seeking employment	160	(3)
Money taken or bank account used coercively	122	(2)
Forced to bring money from natal family	110	(2)
Forced to hand over income	100	(2)
Gambling without her consent	92	(2)
Loan taken without her consent	45	(1)
Any of the above	1106	(23)
Lifetime domestic violence		
Physical violence	1243	(25)
Sexual violence	285	(6)
emotional violence	1553	(32)
Domestic violence in last 12 months		
Physical violence	618	(13)
Sexual violence	186	(4)
Emotional violence	927	(19)
Symptoms of common mental disorders		
Moderate or severe depression on PHQ-9	443	(9)
Moderate or severe anxiety on GAD-7	299	(6)
Suicidal thoughts in the past 12 months	310	(6)
Suicidal action in the past 12 months	31	(< 1)
Suicidal thoughts or action in the past 12 months	314	(6)
All	4906	(100)

*PHQ-9* Patient Health Questionnaire 9-question screen, *GAD-7* Generalised Anxiety Disorder 7-question screen

Table 3 shows crude and adjusted associations between sociodemographic determinants and economic abuse. Economic abuse was more commonly reported by widowed, separated, or divorced women than by married women (aOR 12.4; 95% CI 6.4, 24.1). In adjusted analyses, no clear evidence for a difference in the prevalence of economic abuse was found by age, education, religion, or socioeconomic position, although greater odds were seen for women in remunerated work. Economic abuse was more likely to be reported when husbands used alcohol or drugs (1.2; 1.0, 1.6). In sensitivity analyses, husbands’ use of alcohol or drugs and women’s employment status did not alter the adjusted estimates. Women were more likely to have experienced economic abuse if they had suffered emotional (aOR 6.3; 95% CI 5.0, 7.9), physical (1.9; 1.4, 2.6), or sexual violence (5.4; 3.6, 8.1) in the preceding 12 months.

**Table 3:** Associations of sociodemographic characteristics with economic abuse, for 4906 ever-married women in informal settlements in Mumbai

	Economic Abuse							
	No		Yes					
	n	(%)	n	(%)	OR	(95% CI)	aOR	(95% CI)
Marital status								
Currently married	3710	(79)	984	(21)	1		1	
Widowed, separated, divorced	90	(42)	122	(58)	5.1	(3.7, 7.1)	12.4	(6.4, 24.1)
Respondent age (years)								
18–25	804	(78)	221	(22)	1		1	
26–30	1106	(78)	315	(22)	1.0	(0.8, 1.3)	1.2	(0.9, 1.7)
31–36	915	(78)	257	(22)	1.0	(0.8, 1.3)	1.2	(0.9, 1.6)
37–49	975	(76)	313	(24)	1.2	(0.9, 1.5)	1.5	(1.1, 2.1)
Respondent education (school years)								
No education	726	(77)	212	(23)	1		1	
Primary 1–5	653	(77)	193	(23)	1.0	(0.8, 1.3)	0.8	(0.6, 1.1)
Middle 6–8	830	(76)	269	(24)	1.1	(0.9, 1.4)	0.9	(0.7, 1.1)
High 9–10	844	(76)	261	(24)	1.1	(0.9, 1.3)	1.0	(0.8, 1.3)
Senior 11–12	433	(81)	100	(19)	0.8	(0.6, 1.1)	0.8	(0.6, 1.4)
Above 12	314	(82)	71	(18)	0.8	(0.6, 1.1)	0.9	(0.6, 1.3)
Religion								
Muslim	1403	(77)	423	(23)	1		1	
Hindu	2255	(78)	627	(22)	0.9	(0.8, 1.1)	1.1	(0.9, 1.3)
Other	142	(72)	56	(28)	1.3	(0.9, 1.8)	0.7	(0.4, 1.1)
Caste								
General	2233	(78)	621	(22)	1		1	
Other backward caste	942	(80)	238	(20)	0.9	(0.8, 1.1)	0.8	(0.7, 0.9)
Scheduled tribe or caste	625	(72)	247	(28)	1.4	(1.2, 1.7)	1.3	(1.0, 1.6)
Socioeconomic quintile								
1 poorest	717	(74)	252	(26)	1		1	
2	727	(78)	209	(22)	0.8	(0.7, 1.0)	0.9	(0.7, 1.1)
3	720	(77)	214	(23)	0.9	(0.8, 1.0)	0.9	(0.7, 1.2)
4	727	(78)	206	(22)	0.8	(0.6, 1.0)	0.8	(0.6, 1.1)
5 least poor	765	(82)	170	(18)	0.6	(0.5, 0.8)	0.7	(0.5, 0.9)
Respondent in remunerated work								
No	2982	(80)	742	(20)	1		1	
Yes	818	(69)	364	(31)	1.8	(1.5, 2.1)	1.4	(1.2, 1.7)
Husband in remunerated work								
No	51	(52)	48	(48)	1		1	
Yes	3674	(78)	1012	(22)	0.3	(0.2, 0.5)	0.7	(0.4, 1.3)
Respondent uses alcohol or drugs								
No	3393	(79)	901	(21)	1		1	
Yes	407	(67)	205	(34)	1.9	(1.5, 2.3)	1.2	(1.0, 1.6)
Husband uses alcohol or drugs								
No	2240	(83)	448	(17)	1		1	
Yes	1485	(71)	615	(29)	2.1	(1.8, 2.4)	1.4	(1.2, 1.7)
Emotional violence in last 12 months								
No	3438	(86)	541	(14)	1		1	
Yes	362	(39)	565	(61)	9.9	(8.2, 12.0)	6.3	(5.0, 7.9)
Physical violence in last 12 months								
No	3548	(83)	740	(17)	1		1	
Yes	252	(41)	366	(59)	7.0	(5.4, 8.9)	1.9	(1.4, 2.6)
Sexual violence in last 12 months								
No	3761	(80)	959	(20)	1		1	
Yes	39	(21)	147	(79)	14.8	(10.3, 21.1)	5.4	(3.6, 8.1)

*OR* crude odds ratio for economic abuse, *aOR* adjusted odds ratio for economic abuse, including all covariates in the table

Table 4 shows the associations between economic abuse and screens for depression, anxiety, and suicidal thoughts or actions. Three odds ratios are presented: a crude odds ratio derived from a univariable logistic regression model, an adjusted odds ratio from a multivariable model including sociodemographic covariates, and a further model adjusted with both sociodemographic covariates and covariates describing the other three forms of violence. In the fully adjusted model, economic abuse was associated independently with more than a doubling of the odds of screening positive for moderate or severe depression or anxiety. Physical and sexual violence were associated independently with a 1.5-fold increase in odds. Similar findings were seen for anxiety, and the odds of suicidal thinking or action were more than doubled.

**Table 4:** Associations of economic, emotional, physical, and sexual violence against women with symptoms of depression, anxiety, and suicidal ideation, for 4906 ever-married women in informal settlements in Mumbai

	No	(%)	Yes	(%)	OR	(95% CI)	aOR1	(95% CI)	aOR2	(95% CI)
**Moderate or severe depression on PHQ-9**										
Economic abuse										
No	3441	(95)	165	(5)	1		1		1	
Yes	1022	(79)	278	(21)	5.7	(4.5, 7.1)	4.4	(3.5, 5.4)	2.6	(2.0, 3.4)
Emotional violence in last 12 m										
No	3759	(95)	220	(5)	1		1		1	
Yes	704	(76)	223	(24)	5.4	(4.4, 6.7)	4.8	(3.9, 6.0)	2.5	(1.9, 3.3)
Physical violence in last 12 m										
No	3998	(93)	292	(7)	1		1		1	
Yes	467	(76)	151	(24)	4.4	(3.5, 5.5)	4.0	(3.1, 5.1)	1.4	(1.0, 1.8)
Sexual violence in last 12 m										
No	4336	(92)	384	(8)	1		1		1	
Yes	127	(68)	59	(32)	5.2	(3.8, 7.3)	4.5	(3.1, 6.5)	1.5	(1.0, 2.2)
**Moderate or severe anxiety on GAD-7**										
Economic abuse										
No	3505	(97)	101	(3)	1		1		1	
Yes	1102	(85)	198	(15)	6.2	(4.6, 8.5)	4.8	(3.4, 6.6)	2.7	(1.9, 3.8)
Emotional violence in last 12 m										
No	3843	(97)	136	(3)	1		1		1	
Yes	764	(82)	163	(18)	6.0	(4.6, 7.9)	5.5	(4.1, 7.3)	2.7	(1.8, 4.0)
Physical violence in last 12 m										
No	4099	(96)	189	(4)	1		1		1	
Yes	508	(82)	110	(18)	4.7	(3.5, 6.2)	4.4	(3.2, 6.1)	1.4	(1.0, 2.0)
Sexual violence in last 12 m										
No	4465	(95)	255	(5)	1		1		1	
Yes	142	(76)	44	(24)	5.4	(3.7, 7.9)	4.9	(3.1, 7.8)	1.6	(1.0, 2.6)
**Suicidal thoughts or action in last 12 months**										
Economic abuse										
No	3495	(97)	111	(3)	1		1		1	
Yes	1097	(84)	203	16)	5.8	(4.5, 7.5)	4.9	(3.7, 6. 4)	2.2	(1.5, 3.1)
Emotional violence in last 12 m										
No	3851	(97)	128	(3)	1		1		1	
Yes	741	(80)	186	(20)	7.5	(6.0, 9.5)	6.9	(5.3, 8.9)	2.8	(1.8, 4.2)
Physical violence in last 12 m										
No	4121	(96)	167	(4)	1		1		1	
Yes	471	(76)	147	(24)	7.7	(5.9, 10.1)	6.7	(5.0, 9.1)	2.3	(1.5, 3.5)
Sexual violence in last 12 m										
No	4463	(95)	257	(5)	1		1		1	
Yes	129	(69)	57	(31)	7.7	(5.4, 10.9)	6.2	(4.3, 9.1)	1.8	(1.2, 2.6)

*PHQ-9* Patient Health Questionnaire 9-question screen, *GAD-7* Generalised Anxiety Disorder 7-question screen, *OR* crude odds ratio, *aOR1* odds ratio adjusted with covariates for respondent age, education, religion, caste, socioeconomic quintile, respondent and husband employment, respondent and husband drug or alcohol use, *aOR2* odds ratio adjusted as aOR1 plus covariates for emotional, physical, and sexual violence

**Fig. 1:**
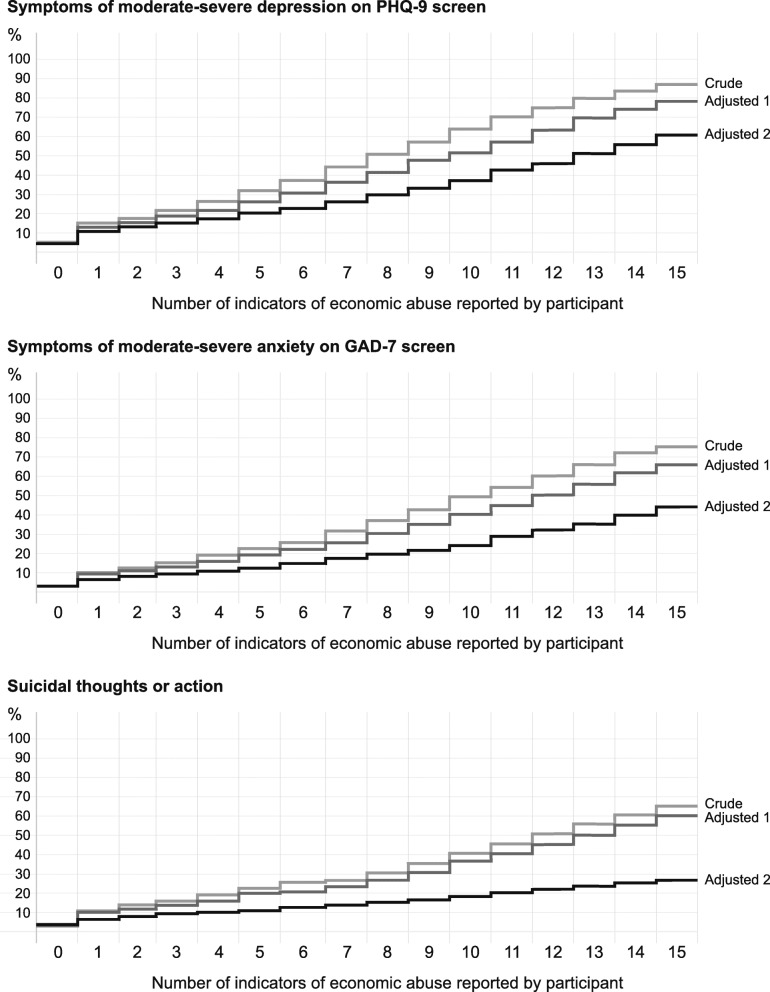
Proportions of women with moderate-severe depression on Patient Health Questionnaire-9 (PHQ-9) screen, moderate-severe anxiety on General Anxiety Disorder (GAD-7) screen, or suicidal thoughts or action, conditional on the experience of 0–15 forms of economic abuse. Adjusted 1: Adjusted with sociodemographic covariates. Adjusted 2: Adjusted with sociodemographic covariates and covariates describing physical, sexual, and emotional violence in the last 12 months

Figure 1 presents the findings from conditional logistic regression models for the impact of economic abuse on positive screens for depression, anxiety, and suicidal ideation. For each outcome, predicted marginal effects are presented for three models: crude, adjusted with sociodemographic covariates, and adjusted with both sociodemographic covariates and covariates describing the three other forms of violence. In the absence of economic abuse, the predicted proportion of women with depression was 5%, with anxiety 3%, and with suicidal thoughts or action 3%. These proportions increased as women reported additional indicators. When women reported 15 indicators of economic abuse, the predicted proportion with depression was 87%% in the crude model (61% in the second adjusted model), with anxiety 76% (44%), and with suicidal thoughts or action 71% (28%). We found no evidence for non-linearity (*p* > 0.05).

## Discussion

### Key findings

In our survey of over 4900 ever-married women aged 18–49 years in informal settlements, 23% had experienced at least one form of economic abuse in their lifetime. The odds of economic abuse were greater when partners used alcohol or drugs and were two-to-five times greater when concurrent emotional, physical, or sexual violence was present. We found little evidence of association with other sociodemographic predictors. Economic abuse was associated with a doubling of reported symptoms of depression, anxiety, and suicidal ideation. The odds of a positive screen for moderate or severe common mental disorders increased with each additional form of economic abuse a woman suffered, a dose-response effect in terms of the breadth of economic abuse.

### Comparison with other studies

To our knowledge, this is the first community-based study in India of the prevalence of economic abuse and its associations with symptoms of common mental disorders. Two strengths were the inclusion of abuse by family members other than intimate partners (important in our context), and the use of established scales to examine associations with mental health symptoms. The questions we used to identify economic abuse were similar to items used in other studies [27, 28, 71, 72], and our findings fell within the reported ranges from India [40–43] and other countries [18, 29, 33, 34]. Economic abuse rarely occurs in isolation and most women who reported it were more likely to report physical, sexual, or emotional abuse [17, 35]. The prevalences of these forms of violence were consistent with previous studies in India [73–77]. In general, the prevalences of depression, anxiety, and suicidal thoughts were comparable with other community studies [78, 79].

### Determinants

Women who were widowed, separated, or divorced were more likely to report experiences of economic abuse in their lifetime. Economic abuse may have contributed to their leaving the relationship: our data did not tell us whether the abuse occurred before or after the split. Nevertheless, past economic abuse can leave a lasting financial burden on survivors [19, 26]. For widowed women, denial of property rights and other means of financial support has been well described in India [80, 81], although age and socioeconomic position did not alter the odds of economic abuse. Women in remunerated employment were more likely to report economic violence than unremunerated women, in accordance with other studies in India which suggest that economic empowerment may not protect women from violence [82], and that violence may increase as partners attempt to compensate for women’s enhanced status and independence associated with employment [83, 84]. Again, it is unclear whether seeking employment was a response to the pressures of a difficult home situation or whether employment increased harassment [85]. Economic abuse keeps women in poverty by reducing their access to independent livelihoods and compromising educational attainment and growth opportunities [26]. The combination of abuse and poverty may trap women in abusive relationships and narrow their focus to basic economic survival. Women’s efforts to become less dependent on the family, or more self-sufficient, have been correlated with an escalation in the intensity and frequency of abuse [85]. The link between alcohol and drug use and domestic abuse is well known [86]. Dependency leads simultaneously to more expenditure on substances, less security of livelihood, and a focus on the availability of money for the user. Alcohol has far-reaching effects on all forms of violence and in initiating and sustaining aggressive behaviour [87].

The large odds ratios illustrated the fact that economic abuse is part of a broader pattern of domestic violence. Our study suggests that economic abuse co-occurred with physical, sexual, and emotional violence, contributing substantially to the totality of violence described recently in rural Bangladesh [29, 50]. Of the 1106 women who reported economic abuse, 51% had also suffered emotional, 13% sexual violence and 33% physical violence. Our finding that economic abuse was more common than physical abuse contrasts with other published estimates [22].

### Associations with symptoms of common mental disorders

Among a range of forms of domestic violence, economic abuse was the strongest predictor of depressive symptoms. The general prevalence of depression was 5%, of anxiety 3%, and of suicidal ideation 4%. With increasing exposure to a range of forms of economic abuse, these figures increased linearly to a cumulative doubling of risk. The literature supports these findings for depression [50, 72], anxiety [25, 34, 88], and suicidal thoughts [89], which have been linked in Indian studies with marital disharmony, domestic violence, harassment by husbands and in-laws, and dowry disputes [90, 91]. Recent work in Bangladesh suggests that economic abuse may account for at least some of the observed associations of other forms of violence with depressive symptoms [50], and it is important that surveys include questions to identify it [29].

Financial stress can lead to a ‘hostile environment’ and psychological distress, and further hamper women’s self-efficacy and capability for independence. The difficulty of breaking the vicious circle of abuse and dependency has been linked with symptoms of common mental disorders and parental difficulties [34].

### Limitations

We did our survey before we began an intervention to prevent violence against women and girls, and we wanted to make sure we sampled women at greatest risk. We also have a commitment to inclusion, and for both these reasons we focused on women with disability and younger married women. We acknowledge that this might have increased the estimated population prevalence of violence. The cross-sectional nature of the study means that causal inference is difficult. The relationship between domestic violence and mental health is bidirectional and mutually reinforcing, and we do not know how much violence followed mental health concerns in either a woman or her abuser. Our study examined past year experience of domestic violence and current mental health, and we could not investigate whether symptoms of common mental disorder led to subsequent reporting of violence or vice-versa. We were also unable to consider factors such as the mental health of partners and family members.

We limited the analytic dataset to married women in order to maximise the applicability of questions about economic abuse for every respondent. However, questions about property rights, seeking employment, and being coerced to hand over income might not have applied to women who had not tried to gain property, jobs, or income. The denominator includes both women who could have encountered the abuse and women who were not in a situation in which it could arise. We think this is a conservative approach in that it tends to reduce rather than increase the estimated prevalence of obstructions to entitlements.

## Conclusion

After more than four decades of research on domestic violence, the burden of economic abuse is becoming clear, although much of the literature comes from high-income countries and women seeking institutional help. Our study provides empirical support for the idea that more subtle forms of domestic violence – such as economic and emotional violence – are at least as harmful to mental health as physical violence. Our study provides evidence that economic abuse is at least as prevalent as physical and emotional abuse that does not happen in isolation but, is part of the constellation of violence against women, and that strongly associated with mental health. Economic abuse and mental health are well recognised issues in India, and the 2005 Prevention of Violence against Women and Girls Act the 2017 National Mental Health Policy are important policy interventions. There is, however, a need to link the two issues. Institutional responses to economic abuse of women are based on limited knowledge of its prevalence, severity, and outcomes and its recognition by care and support providers is essential. Also important is the fact that survivors of economic violence often do not identify it as abuse. We need to create awareness of how abusers exert control over survivors, including efforts to control, exploit, or sabotage employment. Public sector economic development programs should prioritise mechanisms to prevent economic abuse associated with employment and income generation programmes.

## Data Availability

Data are available in the Open Science Framework: Osrin, D. (2020, December 9). Economic abuse dataset. Retrieved from osf.io/jk576.
